# Are patients with hypermobile Ehlers–Danlos syndrome or hypermobility spectrum disorder so different?

**DOI:** 10.1007/s00296-021-04968-3

**Published:** 2021-08-16

**Authors:** Bérengère Aubry-Rozier, Adrien Schwitzguebel, Flore Valerio, Joelle Tanniger, Célia Paquier, Chantal Berna, Thomas Hügle, Charles Benaim

**Affiliations:** 1 Rheumatology, Clinique Bois-Cerf, Lausanne, Switzerland; 2 Rehabilitation and Sports Medicine, Providence Hospital, Neuchâtel, Switzerland; 3 Rheumatology, HFR Fribourg, Villars-sur-Glâne, Switzerland; 4 Physiotherapy, Lausanne University Hospital (CHUV) and University of Lausanne, Lausanne, Switzerland; 5 Center for Integrative and Complementary Medicine & Pain Center, Division of Anesthesiology, Lausanne University Hospital (CHUV) and University of Lausanne, Lausanne, Switzerland; 6 Rheumatology and Rehabilitation, Lausanne University Hospital (CHUV) and University of Lausanne, Lausanne, Switzerland; 7 Department of Medical Research, Clinique Romande de Réadaptation, Sion, Switzerland

**Keywords:** Ehlers–Danlos syndrome, Symptom assessment, Diagnosis-related groups, Outcome assessment, Health care

## Abstract

Diagnosing hypermobile Ehlers–Danlos syndrome (hEDS) remains challenging, despite new 2017 criteria. Patients not fulfilling these criteria are considered to have hypermobile spectrum disorder (HSD). Our first aim was to evaluate whether patients hEDS were more severely affected and had higher prevalence of extra-articular manifestations than HSD. Second aim was to compare their outcome after coordinated physical therapy. Patients fulfilling hEDS/HSD criteria were included in this real-life prospective cohort (November 2017/April 2019). They completed a 16-item Clinical Severity Score (CSS-16). We recorded bone involvement, neuropathic pain (DN4) and symptoms of mast cell disorders (MCAS) as extra-articular manifestations. After a standardized initial evaluation (T0), all patients were offered the same coordinated physical therapy, were followed-up at 6 months (T1) and at least 1 year later (T2), and were asked whether or not their condition had subjectively improved at T2. We included 97 patients (61 hEDS, 36 HSD). Median age was 40 (range 18–73); 92.7% were females. Three items from CSS-16 (pain, motricity problems, and bleeding) were significantly more severe with hEDS than HSD. Bone fragility, neuropathic pain and MCAS were equally prevalent. At T2 (20 months [range 18–26]) 54% of patients reported improvement (no difference between groups). On multivariable analysis, only family history of hypermobility predicted (favorable) outcome (*p* = 0.01). hEDS and HDS patients showed similar disease severity score except for pain, motricity problems and bleeding, and similar spectrum of extra-articular manifestations. Long-term improvement was observed in > 50% of patients in both groups. These results add weight to a clinical pragmatic proposition to consider hEDS/HSD as a single entity that requires the same treatments.

**Supplementary Information:**

The online version contains supplementary material available at 10.1007/s00296-021-04968-3.

## Introduction

Joint hypermobility, the ability to move joints beyond the normal range of motion, is defined as abnormal mobility affecting multiple joints. This health condition can be asymptomatic and has a varied prevalence, 2–57% [1, 2]. In rheumatology clinics, hypermobility is a known risk factor for diffuse musculoskeletal pain. In a few cases, hypermobility is due to heritable disorders of connective tissue, such as osteogenesis imperfecta, Ehlers–Danlos syndrome (EDS), Loeys–Dietz syndrome, Marfan syndrome, and Stickler syndrome [3–5]. In most of these conditions, other organs are involved, which helps the clinician in the diagnosis and in prescribing treatment, especially coordinated programs of reeducation [6–9].

The diagnosis of EDS, especially hypermobile EDS (hEDS), in which extra-articular manifestations can be subtle, is challenging [10]. Misdiagnosis can lead to catastrophic deterioration in health. In 2017, a revised classification of EDS subtypes was published [11]. These subtypes share a common phenotype: the presence of hypermobility, hyperlaxity of the skin, and fragility of several tissues. On a molecular level, identification of a causative variant(s) in the respective gene is possible in 12 subtypes. Despite a probable autosomal dominant inheritance [11] and reports of haploinsufficiency or missense of tenascin X in a few cases [12, 13], no gene has been identified for hEDS [11, 14].

In the absence of molecular support, in 2017, the International EDS Consortium proposed new diagnosis criteria for hEDS [11, 15] that were based on a set of clinical criteria, expertise of the clinicians, and absence of another pathology explaining the symptoms of hEDS. Patients with symptomatic syndromic joint hypermobility but not fulfilling the new diagnostic criteria for hEDS are characterized as having hypermobility spectrum disorder (HSD). Unfortunately in recent cohort publications [16, 17], these new criteria seemed to not adequately identify the more severely affected patients and did not highlight some extra-articular manifestations of hEDS such as bone involvement [18], neurologic involvement (including small-fiber neuropathy [19]), sleep disorders [20], and immune system disorders such as mast cell disorders [21]. Moreover, we do not know whether patients with the 2017 hEDS diagnostic criteria have more or less chance of responding to adequate management, including rehabilitation, as compared with those with HSD. Thus, some authors rapidly raised their limits and proposed to group these conditions in a single phenotype, termed hEDS/HSD [17](https://www.ehlers-danlos.com/2017-eds-international-classification/) to provide good management and treatment for all of them. In the same vein, very recent studies consider that hEDS and HSD exist on the same physiological continuum (hEDS being a more marked form) [22], require the same pattern of multidisciplinary intervention [23, 24], or are gathered in the same category for research purposes [25, 26].

The main aim of our study was to compare patients classified as having hEDS and HSD according to the 2017 diagnostic classification in terms of overall severity of clinical symptoms including 3 extra-articular manifestations: bone involvement, neuropathic pain complaints and symptoms of mast cell disorders. The secondary objective was to compare the patients’ mid-term evolution after undergoing standardized coordinated physical therapy management for at least 1 year.

## Methods

### Study design and setting

All patients attending the hypermobility-dedicated consultation at a single Swiss reference center (Bone and Joint Department, Lausanne University Hospital, Lausanne, Switzerland) between November 2017 and April 2019 could be included in this real-life prospective cohort. Rheumatologists, physiatrists, physiotherapists, occupational therapists, and clinical geneticists composed the team.

### Participants

#### Inclusion criteria

**Fig. 1:**
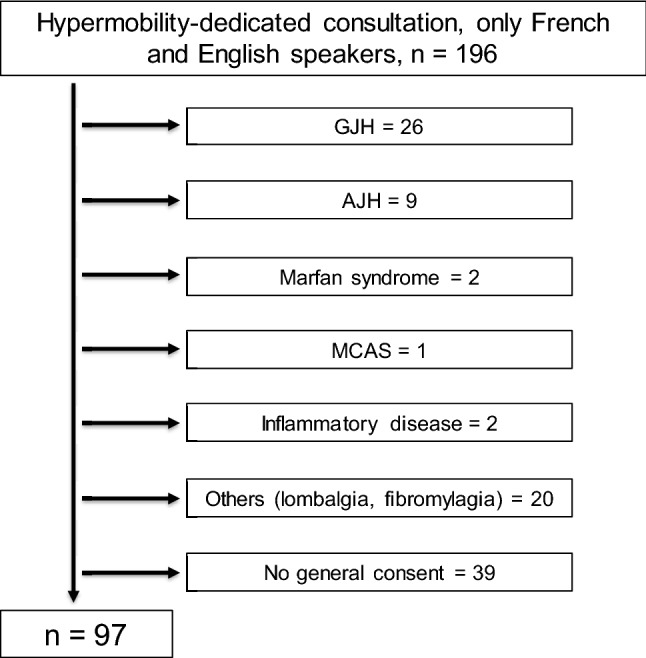
Patients flow chart. GJH: generalized joint hypermobility, AJH: asymptomatic joint hypermobility, MCAS: mast cell activation syndrome

Adult patients (≥ 18 years of age) with (Fig. 1) symptomatic generalized hypermobility syndrome who have signed the general consent for research in our institution were included. The Lausanne University Hospital informs all patients about further use of biological material and clinical data for research purposes and proposes them to fill in a general consent form (General Consent For Research). The present study solely includes patients who have given their agreement to this General Consent.

#### Exclusion criteria

- Patients with another diagnosis explaining their articular symptoms or syndrome.

- Non-French or -English speakers.

### Ethics

The local ethical committee (Commission cantonale (VD) d'éthique de la recherche sur l’être humain (CER-VD)) approved this protocol in 2012 (ID project PB_2019-00,098 (144/12) and the use of the General Consent Form for research.

### Variables

#### The 2017 diagnostic criteria [11] (supplementary file)

To be classified as having hEDS, patients had to meet the following 3 criteria; otherwise, they were classified as having HSD: 1) generalized joint hypermobility, based on the Beighton score: “Yes” =  > 6 pre-puberty, > 5 up to age 50, > 4 after age 50, > 5 anamnestic; 2) presence of at least 2 of the following 3: A = at least 5 systemic manifestations (see list in the supplementary file 1); B = positive family history; C = musculoskeletal involvement; and 3) exclusion of other diagnoses explaining the symptoms. In the new 2017 diagnostic criteria, bilateral piezogenic papules of the heel were added to criterion 2A, which justified recording this symptom separately.

#### Symptom severity

The Beighton score was used in its quantitative form (0–9) for analysis. To evaluate symptom severity, we constructed a subjective score based on the 16 clinical items of a questionnaire (Clinical Severity Score 16 [CSS-16]: pain, fatigue, sleep disturbance, motricity problems, skin, dysautonomia, cardiac, spontaneous bleeding, gastrointestinal, bladder, temporomandibular joint, ear–nose–throat [ENT], visual, lung tract, sexual and cognitive involvements) (Table 1), each rated on a Likert scale from 0 to 4 (0, no manifestation; 1, minor; 2, moderate; 3, severe; 4, very severe), with total score 0 to 64. This scale was inspired by the work of Hamonet et al. [27] The scale has not been validated in terms of sensitivity and specificity for hESD/HSD but pragmatically reflects our clinical evaluation. The CSS-16 questionnaire was self-administered, assisted by a physician. This questionnaire is a list of symptoms and is only available in French. For patients who understand English only, the examiner orally lists the symptoms in English and the patient gives his or her evaluation for each symptom.

**Table 1:** Description of the phenotype hypermobile Ehlers–Danlos syndrome (hEDS) versus hypermobile spectrum disorders (HSD)

	hEDS (*n* = 61)	HSD (*n* = 36)	*p* value
Age	40.0 (31.0–52.0)	39.0 (30.0–48.0)	0.82
Sex m/f	4/57	3/33	0.74
Beighton score (0–9)	7.0 (6.0–9.0)	7.0 (4.0–8.0)	0.03*
2017 Classification, criterion 1 +	90%	71%	0.07
2017 Classification, criterion 2 + 2A + 2B + 2C +	100% 18% 93% 98%	27% 0% 33% 75%	< 10^–4^ < 10^–2^ < 10^–4^ < 10^–3^
2017 Classification, criterion 3 +	100%	78%	< 10^–4^
Piezogenic papules	29%	22%	0.30
CSS-16 (total score 0–64)	40.0 (31.0–52.0)	31.5 (22.3–35.0)	0.04*
Pain (0–4)	3.0 (3.0–4.0)	3.0 (2.0–4.0)	0.05*
Fatigue (0–4)	4.0 (3.0–4.0)	3.0 (3.0–4.0)	0.41
Sleep disturbance (0–4)	3.0 (2.0–4.0)	2.0 (1.3–3.0)	0.15
Motricity problem (0–4)	4.0 (3.0–4.0)	3.0 (2.3–3.0)	0.01*
Skin problem (0–4)	2.0 (1.0–3.0)	1.5 (1.0–2.8)	0.20
Dysautonomia (0–4)	2.0 (2.0–3.0)	2.0 (2.0–3.0)	0.75
Cardiac problem (0–4)	0.0 (0.0–0.0)	0.0 (0.0–1.0)	0.55
Bleeding (0–4)	2.0 (1.0–3.0)	2.0 (1.0–2.0)	0.05*
GI problem (0–4)	3.0 (1.5–3.0)	2.5 (1.0–3.0)	0.41
Bladder problem (0–4)	2.0 (0.0–3.5)	2.0 (0.0–2.8)	0.23
TMJ problem (0–4)	2.0 (0.0–3.0)	2.0 (1.0–2.0)	0.70
ENT problem (0–4)	2.0 (1.0–3.0)	2.0 (1.0–2.8)	0.36
Visual problem (0–4)	2.0 (0.5–3.0)	2.0 (1.0–2.0)	0.29
Lung tract problem (0–4)	2.0 (0.0–3.0)	1.5 (0.0–2.8)	0.73
Sexual problem (0–4)	1.0 (0.0–2.0)	1.0 (0.0–2.0)	0.96
Cognitive problem (0–4)	2.0 (1.0–3.0)	1.0 (0.0–2.8)	0.11
Bone fragility	23%	28%	0.63
DN4 + (> 4/10)	47%	49%	1.00
Suspected MCAS	43%	50%	0.54

Data are reported as % or median (percentiles 25th-75th). *m/f* male/female. *GI* gastrointestinal. *TMJ* temporomandibular joint. *ENT* ear–nose–throat. *MCAS* mast cell activation syndrome. *CSS-16* Clinical Severity Score of 16-item questionnaire. *DN4* Douleur Neuropathique 4 (positive if > 4/10)

^*^Statistically significant

#### Extra-articular manifestations (bone, neuropathic pain and mast cell disorders)

- Bone involvement was assessed by a question assessing the prevalence of non-traumatic fractures and personal history of low bone mineral density (BMD).

- Neuropathic pain, with underlying suspected small-fiber neuropathy, was based on a pain detection score (Douleur Neuropathique 4 [DN4]) > 4/10 in at least 2 extremities [28].

- Mast cell activation syndrome (MCAS) was clinically suspected if the patient reported the coexistence of flush and/or dermographia.

### Assessment schedule

**Fig. 2:**
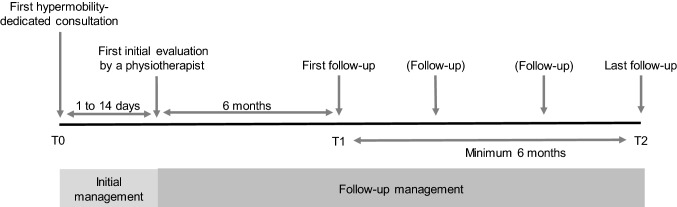
Timeline of the study

All patients underwent an (Fig. 2) initial medical assessment at baseline (T0), an interim medical assessment at 6 months (T1), then at least one medical assessment at ≥ 12 months (T2). Data collected at T0 were the 2017 diagnostic classification, CSS-16 score and clinical detection data for bone involvement, neuropathic pain and MCAS. On T1 and T2, patients were asked if they considered their condition improved or not. Only data on T2 were used to evaluate the mid-term evolution.

### Management

Shortly after the medical assessment, patients underwent a specialized physical therapy evaluation from our team. This assessment allowed to establish a treatment plan for physical therapy management. Regardless of the diagnosis of hEDS or HSD, the treatment included physical therapeutic approaches aimed at body awareness/proprioception as well as low-resistance/low-impact exercises in a closed or semi-closed kinetic chain to strengthen deep and stabilizer muscles. A plan for home-based exercises was established and included a cognitive behavioral approach. Patients were encouraged to re-start progressive physical activity or sports and were given some recommendations for specific activities. At T1 and T2, patients were asked to consider pain, disability, injury and empowerment together in answering the following question: “Compared to the initial assessment, how do you now consider yourself regarding pain, disability, injury and empowerment?”, with possible answers of improved/not improved.

### Statistical analysis

Patient characteristics are reported as frequency (percentage) or median (percentiles 25th–75th) for categorical and continuous variables, respectively. To test the ability of the 2017 diagnostic criteria to identify patients with clinically severe disease and with extra-articular involvement, we compared CSS-16 scores and prevalence of bone involvement, neuropathic pain and MCAS symptoms between the hEDS and HSD groups. We also investigated the effect on T1 and T2 outcomes of the 2017 diagnostic criteria, CSS-16, family history, bone involvement, neuropathic pain, and MCAS symptoms.

Associations between studied parameters were tested with Mann–Whitney U-Test and chi-square test or Fisher exact test as appropriate. After univariate tests to determine which factors significantly (*p* < 0.10) affected outcome at T2 (improved/not improved), the best predictors were tested on multivariable logistic regression analysis (*p* ≤ 0.05). Statistical analyses involved using Stata ICv14 for Windows (StataCorp, College Station, TX, USA). *P* ≤ 0.05 was considered statistically significant.

## Results

### Participants

Between November 2017 and April 2019, 196 patients attended the dedicated hypermobility consultation in the rheumatology unit; 97 patients were included in the final analysis (Fig. 1). Overall, 61 patients fulfilled the hEDS 2017 diagnostic criteria and 36 did not (HSD group). The characteristics of the two groups are summarized in Table 1. The median age was 40 (30–50, range 18–73) and 92.7% were females, with no between-group differences.

### Outcome data: 2017 diagnostic criteria

The Beighton score was significantly (Table 1) higher for hEDS than HSD patients (7.0[6.0–9.0] vs. 7.0[4.0–8.0], *p* = 0.03) but not in its categorical form (no/yes: < 5/ > 5) according to 2017 criterion 1. Criteria 2017 “2” and “3” were by definition more often present in hEDS than HSD patients: the proportion of > 5 systemic manifestations (criterion 2A) was 18% and 0% (*p* < 10^–2^), positive family history (2B) 93% and 33% (*p* < 10^–4^), and musculoskeletal pain or chronic or recurrent dislocations (2C) 98% and 75% (*p* < 10^–3^). In the HSD group, 22% had another diagnosis that could be confounding with symptoms (Fabry disease, psoriasic arthritis, multiple sclerosis, Sjögren syndrome). The groups did not differ in presence of bilateral piezogenic papules of the heel (hEDS and HSD: 29% vs 22%, *p* = 0.30).

### Symptom severity and extra-articular manifestations: bone, neuropathic pain, mast cell disorders

All included patients reported pain, (Table 1) most (82%) with a severe score (≥ 3/4). Fatigue, sleep disturbance, dysautonomia and gastrointestinal symptoms were severe in > 40% of patients. CSS-16 scores were significantly higher for hEDS than HSD patients (40.0[31.0–52.0] vs. 31.5[22.3–35.0], *p* = 0.04), but among the 16 items, only pain, motor and bleeding problems were significantly more severe in hEDS than HSD patients (Table 1 and Supplementary file).

The proportion of patients with anamnestic bone fragility was 23% and 28% in the hEDS and HSD groups, DN4 score > 4 was 47% and 49%, and suspected MCAS 43% and 50%, with no significant difference.

Spontaneous bleeding problems were significantly more severe in patients with than without suspected MCAS (2.0[2.0–3.0] vs. 2.0[0.3–2.0], *p* < 10^–3^).

### Follow-up data: evolution with standardized physical therapy management

Follow-up data were available for 76 patients at T1 (78%) and 59 at T2 (61%), with a mean follow-up time of 6(6–10) and 20(18–26) months, respectively. Patients lost to follow-up at T1 did not differ from others in age (*p* = 0.29), sex (*p* = 0.64) or Beighton score (*p* = 0.26). However, patients lost to follow-up at T1 had a lower CSS-16 score than those not lost to follow-up (28.0[18.5–34.5] vs. 34.0[27.0–40.5], *p* = 0.016) and the proportion of lost patients was lower in the hEDS than HSD group (13% vs. 36%, *p* = 0.01). The situation was comparable at T2: same age (*p* = 0.21), sex (*p* = 1.00) and Beighton score (*p* = 0.23). Patients lost to follow-up at T2 had a slightly lower but not significantly CSS-16 score than those not lost to follow-up (32.5[24.0–35.3] vs*.* 34.0[27.0–41.0], *p* = 0.12) and the proportion of lost patients was lower in the hEDS than HSD group (30% vs. 56%, *p* = 0.02).

**Fig. 3:**
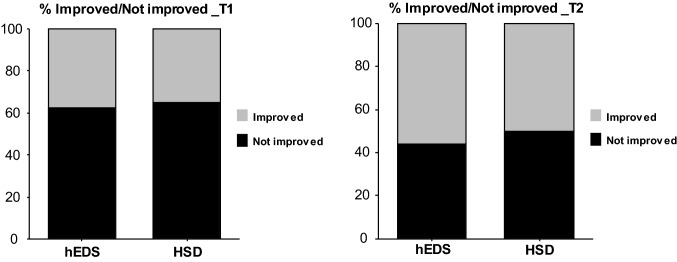
Evolution of condition at T1 (6 months) and T2 (end of follow-up). No difference between groups

In total, 28 (36.8%) patients considered their condition improved at T1 and 32 (54%) at T2 (Fig. 3). The slight percentage difference in improvement favoring the hEDS group was not significant (T1: 38% vs. 35%, *p* = 1.00; T2: 56% vs. 50%, *p* = 0.77).

On univariate analyses, factors associated with better prognosis at T2 were the initial intensity of pain (*p* = 0.05), sleep disturbance (*p* = 0.06), family history of hypermobility (*p* = 0.07) and DN4 positivity (*p* = 0.04) (Table 2). On multivariate analysis, only family history of hypermobility was an independent predictor of (favorable) outcome (*p* = 0.01).

**Table. 2:** Baseline predictors of evolution at end of follow-up (T2) (*n* = 59). Data are reported as % or median (percentiles 25th–75th)

	Amelioration of condition	No amelioration of condition	*p* value (univariate)	*p* value (multivariate)
Pain	3.0 (3.0–4.0)	4.0 (3.0–4.0)	*p* = 0.05*	*p* = 0.26
Fatigue	3.0 (3.0–4.0)	4.0 (3.0–4.0)	*p* = 0.15	
Sleep problem	2.0 (1.0–4.0)	3.0 (2.0–4.0)	*p* = 0.06*	*p* = 0.18
Motricity	3.5 (2.0–4.0)	4.0 (3.0–4.0)	*p* = 0.38	
Skin	2.0 (1.0–3.0)	2.0 (1.0–3.0)	*p* = 0.48	
Dysautonomia	2.0 (2.0–3.0)	3.0 (2.0–3.0)	*p* = 0.32	
Cardiac	0.0 (0.0–1.0)	0.0 (0.0–0.0)	*p* = 0.41	
Bleeding	2.0 (1.3–3.0)	2.0 (1.0–3.0)	*p* = 0.11	
GI	3.0 (2.0–3.0)	3.0 (1.0–3.0)	p = 0.38	
Bladder	2.0 (0.3–3.0)	2.0 (1.0–4.0)	*p* = 0.79	
TMJ	2.0 (0.0–3.0)	1.0 (0.0–3.0)	*p* = 0.73	
ENT	2.0 (1.0–3.0)	1.0 (0.0–3.0)	*p* = 0.52	
Visual	2.0 (1.0–3.0)	2.0 (0.0–3.0)	*p* = 0.71	
Lung tract problem	2.0 (1.0–2.8)	2.0 (0.0–3.0)	*p* = 0.53	
Sexual	0.5 (0.0–2.0)	1.0 (0.0–2.0)	*p* = 0.77	
Cognitive	2.0 (0.0–2.0)	2.0 (1.0–4.0)	*p* = 0.22	
Family history			*p* = 0.07*	*p* = 0.01**
No	22.22%	77.78%		
Yes	58.70%	41.30%		
Bone fragility			*p* = 0.77	
No	53.49%	46.51%		
Yes	60.00%	40.00%		
DN4 +			*p* = 0.04*	*p* = 0.12
No	68.75%	31.25%		
Yes	40.00%	60.00%		
Suspected MCAS			*p* = 0.43	
No	50.00%	50.00%		
Yes	61.54%	38.46%		

^*^Statistically significant p < 0.10 (univariate). **Statistically significant *p* < 0.05 (multivariate). *GI* gastrointestinal. *TMJ* temporomandibular joint. *ENT* ear-nose-throat. *MCAS* mast cell activation syndrome. *DN4* Douleur Neuropathique 4 (positive if > 4)

## Discussion

This study provided additional information about the limited accuracy of the new 2017 diagnostic criteria to distinguish the severity of symptoms between patients with a diagnosis of hEDS and HSD and the prevalence of extra-articular manifestations: detection of bone fragility, neuropathic pain and MCAS symptoms. The results highlighted the possibility to add another more specific symptom to hEDS: severity of spontaneous bleeding. This study showed that a coordinated physical therapy program could improve patient symptoms similarly in hEDS and HSD, for about half of the patients.

### More pain, disability, and spontaneous bleeding in hEDS than HSD patients, but the 2017 diagnostic criteria were not related to other disabling manifestations

In our cohort, hEDS patients presented a more severe phenotype than did HSD patients: significantly more pain, disability and hypermobility. These results were not surprising. Per the definition, patient disability is related to the Beighton score (diagnostic criterion 1) and pain to criterion 2C: musculoskeletal involvement. However, we found a significantly higher prevalence of severity of spontaneous bleeding in hEDS than HSD patients. Traditionally, the bleeding symptom was associated with the vascular form of EDS. However, patients with abnormal hypermobility experience more abnormal bleeding [29]. The bleeding is related to platelet dysfunction or mast cell activation. Our patients showed a high prevalence of symptoms suggesting MCAS. Nevertheless, this symptom of spontaneous bleeding was not considered sensitive and specific enough to be included in the 2017 diagnostic criteria [11].

The 2017 criteria were proposed to avoid neglecting a potentially severe disorder and to limit overemphasizing a non-pathological variation from the norm [15]. Thus, the 2017 criteria of hEDS limits the diagnosis to patients with clear Mendelian transmission or those with extra-articular and systemic manifestation. Yet, some authors doubt that the more severely affected patients are correctly identified [16, 17, 30, 31]. In our study, with our severity score, we confirmed that more severely affected patients (more pain or disability) were well detected by the 2017 diagnostic criteria. Nevertheless, Copetti et al. [17] (105 patients, 58 hEDS) and Mc Gillis et al. [16] (131 patients, 10 hEDS), found the opposite: the distinction between hEDS and HSD diagnosis based on the 2017 diagnostic criteria did not reveal any differences in severity when defined based on the intensity of pain, autonomic symptoms, functional difficulties, fatigue, attention deficit and quality of life.

We recorded piezogenic papules separately, added as a diagnostic feature in 2017 [11], based on a small study [32]. As for Mc Gillis et al. [16], piezogenic papules were no more frequent in hEDS than HSD patients in our larger hEDS cohort. Thus, our study found that patients with a diagnosis of hEDS did not have a significantly more severe phenotype than HSD patients.

### hEDS and HSD patients showed a high prevalence of extra-articular involvement: suspected bone involvement, neuropathic pain or mast cell disorders

The 2017 diagnostic criteria could not distinguish patients with more extra-articular manifestations, bone fragility, neuropathic pain, or suspected MCAS. The prevalence of anamnestic bone fragility was high (23% for a median age of 40) in our cohort, when considering that in the normal population of women aged ≥ 50 years in Europe, the prevalence of osteoporosis is 22.5% [33]. We recorded bone fragility as a non-trauma fracture or BMD value lower than normal value. The Eller-Vainicher et al. [18] study of 50 Caucasian patients with hEDS or classical EDS (diagnosed with the older criteria of hEDS), mean age 40.3 ± 5.9 years, 72% women, reported a prevalence of 32% of bone fragility. The authors evaluated bone health based on bone quantity (with BMD measured by dual x-ray absorptiometry) and bone quality (evaluated by Trabecular Bone Score) in addition to the detection of vertebral fracture (screened with conventional spinal radiography in lateral and anteroposterior projection T4–L4 assessment). In a review, Formenti et al. [34] proposed to screen all patients with hEDS by dual X-ray absorptiometry. Finally, Banica et al. [35] suggested that bone fragility in hEDS or HSD patients could be linked to lower mechanical strain. None of these prior studies suggested a difference between hEDS and HSD patients, but a high prevalence of bone involvement seems confirmed.

The neuropathic pain detection score was frequently positive in our cohort, almost 50% in both groups. Chronic pain is an important problem for HSD/hEDS patients [36]. Neuropathic pain has been described as related to small-fiber neuropathy in HSD/hEDS [19] as well as to the well-known nerve luxation/subluxation related to the hypermobility. A diagnosis is important because the therapeutic approach is different: medication for primary neuropathic pain versus proprioceptive control or surgery for hypermobility-related pain.

In approximately 45% of patients, symptoms were compatible with MCAS. The GoodHope study [16] found the same prevalence of MCAS in both hEDS and HSD groups, but approximately only 25%. Since this publication, other articles reported a link between hEDS and MCAS [37, 38], and patient EDS association reported this possible association (https://www.ehlers-danlos.com). Therefore, it could be a bias of over-positivity of MCAS symptoms in our cohort. As suggested by Jesudas et al. [29], we found a positive association between spontaneous bleeding and suspected MCAS.

### Coordinated physical therapy management could improve symptoms similarly in both patient groups

In our study, we proposed the same management for hEDS and HSD patients based on an initial assessment by a physiotherapist and a semi-standardized reeducation program, coached by a physiotherapist and then progressively trusted to the patients themselves (self-care). Strong evidence for physical therapy is lacking [39], yet it is the mainstay of management [8, 40, 41]. Generally, to improve, treat and prevent musculoskeletal manifestations of joint hypermobility, the facets of education, active participation and active physical therapy intervention are recommended. [23, 24, 40–43]. Hope et al. [44] showed that all hEDS and HSD patients had higher frequency and severity of subjective health complaints than matched controls. The main explanation was low understanding of the patient’s illness and associated symptoms and moderate beliefs that the illness could be kept under control through self-management, reeducation or treatment.

With our program, more than 50% of patients showed improved articular symptoms at the end of follow-up. In 2013, Bathen et al. [45] showed improvement in perceived performance of daily activities, muscle strength and endurance in 12 women via a cognitive behavioral-based intervention including teaching easy exercises to perform at home. We also pragmatically based our program on the need to perform the rehabilitation at home but included a more ambitious step in our clinic to further reassure and empower our patients.

The diagnostic category (hEDS vs. HSD) does not appear to be a prognostic factor for outcomes after physical therapy. Good clinical practice for hEDS and HSD must integrate a coordinated physical therapy program, if possible within a network of experienced caregivers, which could become the standard of care. The only factor that seemed to influence a favorable evolution was family history of hypermobility. We have no straightforward explanation for this finding. Perhaps, empowerment is facilitated by the presence of the disability in a parent or a child in the same family. Also, having another family member with the same diagnosis could induce motivation for rehabilitation.

Among patients lost to follow-up, we found lower CSS-16 scores and a higher proportion of HSD diagnoses than hEDS. We can reasonably assume that the severity of symptoms and a clear diagnosis are motivational factors for attending a tertiary center.

### Strengths and limitations

Being the only coordinated center in the French speaking part of Switzerland, our sample is representative of the hypermobile and hEDS/HSD patients in this region with a global population of 2 million. Reassuringly, the proportion of women [16, 17], mean age, prevalence of pain, and fatigue are similar to that in prior studies [16, 17, 40]. With this dataset, we can confirm that hEDS is not rare [46]: 42% of the patients referred to the hypermobility-dedicated consultation met the 2017 diagnostic criteria. Furthermore, for the first time, a coordinated physical management program resulted in improvement in slightly more than 50% of patients. These results are encouraging and motivating when we know that instability is the main cause of pain and deteriorated quality of life in hypermobile patients.

Our study has limitations. This was a monocentric study in a small country, yet as described above, it is the referral center, which holds its importance. The scores used were mostly subjective or detection tools, which are convenient to use in a clinical setting. The CSS-16 is not validated, and its sensitivity and specificity for hEDS or compared to other disorders such as fibromyalgia is not known. A validation and comparison with other cohorts is needed, given the utility and easy application of this score. The global evolution is a composite self-report regarding pain, disability, injury and empowerment. We did not record evolution reports for these elements separately, and therefore could not study whether pain was decreased, for example. This plan was chosen on the basis of clinical relevance (global function favored over individual item scores). Unfortunately, we were not able to assess the reason for lost to follow-up because it was the patients’ decision not to return to clinical care, and it would have been invasive to ask them the reason for this. The reason could be a bias in the results we present (underestimation of effects with more loss of patients with great improvement or over-estimation with more loss of patients with deterioration).

### Conclusion

Based on a clinical severity scale of 16 items, in our cohort, patients with hEDS fulfilling the 2017 diagnostic criteria and HSD patients showed globally similar severity scores except for pain, motricity problems and spontaneous bleeding, and similar spectrum of extra-articular manifestations. In addition, improvement was ≥ 50% with a coordinated physical therapy program in both groups. Altogether, these results add weight to the proposition to consider hEDS/HSD as a single entity that requires the same treatments.

## Supplementary Information

Below is the link to the electronic supplementary material.

Supplementary file1 (PDF 22 KB)
