# Supplementary figures and images for: Are patients with hypermobile Ehlers–Danlos syndrome or hypermobility spectrum disorder so different?
Source: Rheumatol Int. 2021 Aug 16;41(10):1785–94. doi: 10.1007/s00296-021-04968-3 (PMC8390400; doi:10.1007/s00296-021-04968-3)

## hEDS

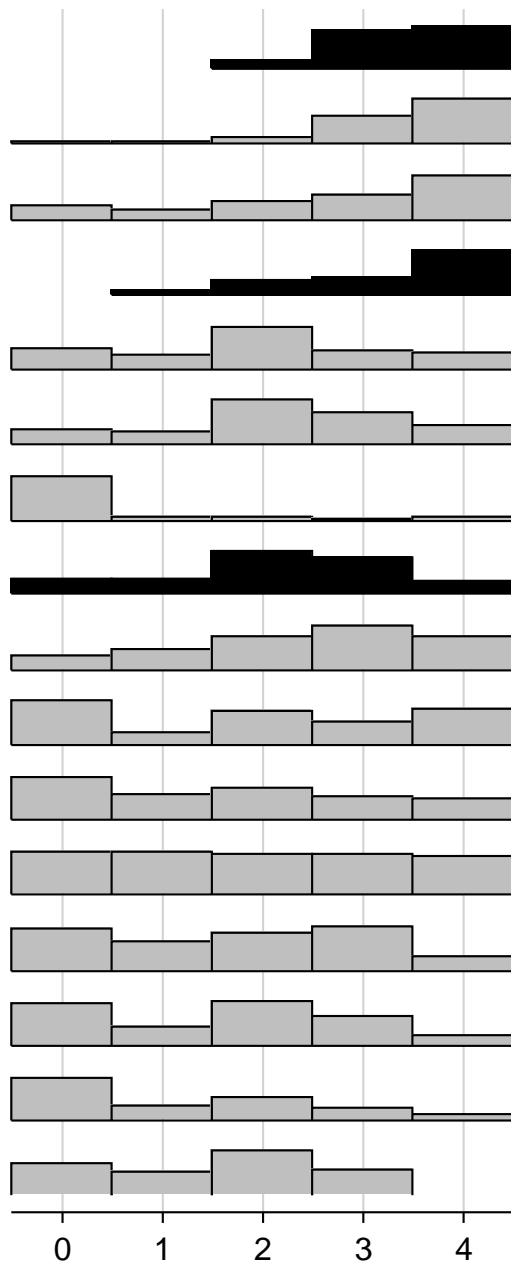

## HSD

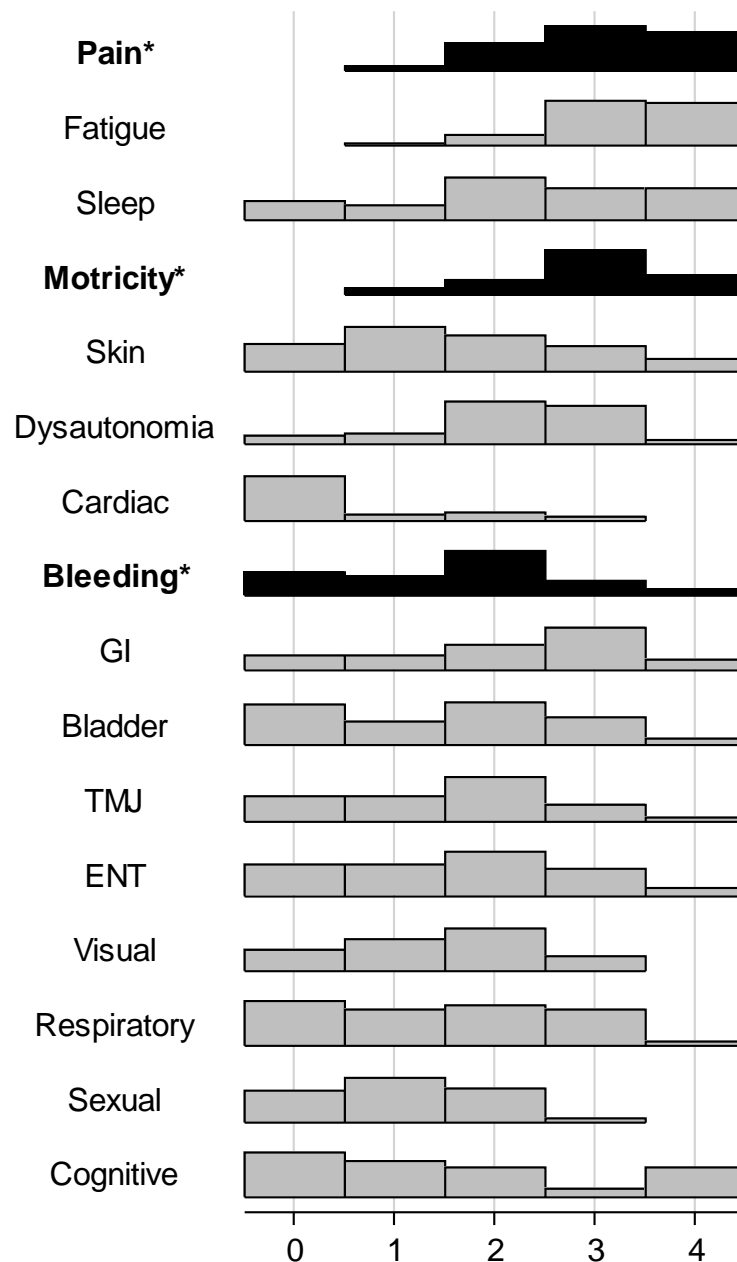

Supplement: Supplementary file 1 — Supplementary file1 (PDF 22 KB) [file 296_2021_4968_MOESM1_ESM.pdf]
